# Impact of Preoperative Hospital Stay on Postoperative Complications in Elective Neurosurgical Procedures: A Retrospective Observational Study in a Tertiary Care Hospital in Nepal

**DOI:** 10.1002/hsr2.73462

**Published:** 2026-11-10

**Authors:** Mohan Raj Sharma, Ishan Sharma, Susmin Karki, Nirav Ojha, Khusbu Kumari, Prakash Regmi, Deepak Raj Joshi, Gopal Sedain, Sushil Krishna Shilpakar

**Affiliations:** ^1^ Department of Neurosurgery Tribhuvan University Institute of Medicine Kathmandu Bagmati Nepal; ^2^ Nepal Medical College Kathmandu University Kathmandu Bagmati Nepal; ^3^ Maharajgunj Medical Campus Tribhuvan University Institute of Medicine Kathmandu Bagmati Nepal; ^4^ Department of Community Medicine and Public Health Maharajgunj Medical Campus Kathmandu Bagmati Nepal

**Keywords:** neurosurgery, postoperative complications, preoperative hospital stay

## Abstract

**Background and Aims:**

Preoperative stay varies with patient and institutional factors. In Neurosurgery, at least 1 day of admission before any procedure is usually the norm. This study evaluates the correlation between preoperative length of stay (LOS) and postoperative complications, classified by the Clavien–Dindo classification, among patients undergoing elective neurosurgical procedures at a university hospital in Nepal.

**Methods:**

We retrospectively reviewed records of 110 patients who underwent elective neurosurgery from June 2022 to June 2023. Data included demographics, comorbidities, preoperative LOS, total LOS, diagnosis, treatment, postoperative complications, and 3‐month outcomes.

**Results:**

Of 110 patients, 54.5% (60/110) were male, with a median age of 35 years. Most (78.2%) had no comorbidities; hypertension was the most common (10.9%). The median total hospital stay was 13 days, with a median preoperative stay of 5.5 days. Postoperatively, 80.0% of patients required at least 1 day in the intensive care unit. The incidence of postoperative complications was 33.6% (37/110). Complications occurred in 33.6% of patients. Preoperative LOS was significantly associated with postoperative complications (*p* = 0.045) but not with Clavien–Dindo grade (*p* = 0.063). Total LOS was strongly associated with both postoperative complications (*p* < 0.001) and complication severity by Clavien–Dindo classification (*p* < 0.001).

**Conclusion:**

Longer preoperative and total hospital stays were associated with increased postoperative complications, while total LOS was also linked with complication severity. These findings emphasize the influence of preoperative hospitalization on surgical outcomes. Optimizing preoperative management and minimizing unnecessary delays may reduce morbidity following neurosurgical procedures.

**Abbreviations**
ASA: American Society of Anesthesiologists
AVM: arteriovenous malformation
CD: Clavien–Dindo (classification system)
CI: confidence interval
CNS: central nervous system
COVID: coronavirus disease 2019
CSF: cerebrospinal fluid
ICU: intensive care unit
IQR: interquartile range
IV: intravenous
LOS: length of stay
MMC: meningomyelocele
OR: odds ratio
SAMPL: Statistical Analyses and Methods in the Published Literature
SD: standard deviation
SPSS: Statistical Package for the Social Sciences
SSI: surgical site infection
STROCCS: strengthening the reporting of cohort studies in surgery
TUTH: Tribhuvan University Teaching Hospital
US: United States

## 1 Introduction

Neurosurgical diseases that require surgery are often associated with significant morbidity and mortality [1]. The length of stay (LOS) in neurosurgery depends on the patient's primary diagnosis, the presence of comorbidities [2] requiring further work‐up to optimize for surgery, the degree of access to operating rooms, and the urgency of the procedure [3]. Our institution admits patients 1 day before the scheduled procedure, as most preoperative workups are performed on an outpatient basis. This practice allows for a minimal duration of bed occupancy and lowers the financial burden on the patient, and reduces the risk of hospital‐acquired infection. According to the Clavien–Dindo system, postoperative complications are classified into five categories [4]. The most frequent complications are bleeding and re‐operation within 30 days of the initial operation [5]. Neurosurgery‐specific complications include seizures, cerebral edema, and ischemia [6]. The impact of preoperative hospital stay on postoperative complications in elective neurosurgical procedures is multifaceted, influenced by surgery type, patient factors, socioeconomic constraints, and healthcare system structure, particularly in low‐income countries. The length of preoperative hospital stay has been associated with an increased risk of surgical site infections (SSIs) in neurosurgical procedures. A study conducted in China found that longer preoperative stays were significantly associated with higher SSI prevalence, particularly deep/space‐organ SSIs, after adjusting for confounders such as the American Society of Anesthesiologists score and type of surgery [7]. This suggests that prolonged preoperative hospitalization may contribute to increased postoperative complications, potentially due to increased exposure to hospital pathogens. In contrast, a study from Switzerland did not find a significant independent association between preoperative LOS and SSI risk after adjusting for confounders, although preoperative admission to an intensive care unit (ICU) was associated with increased odds of SSI [8]. This indicates that the relationship between preoperative stay and postoperative complications may vary based on healthcare settings and patient management practices.

Factors such as patient age, functional status, and hospital‐acquired infections have been identified as contributors to prolonged hospital stays in elective neurosurgery, which can indirectly affect postoperative outcomes [9]. Additionally, preoperative assessments, including ASA scores and C‐reactive protein levels, have been shown to predict in‐hospital complications and resource use, highlighting the importance of comprehensive preoperative evaluation [10].

In low‐income countries like Nepal, socioeconomic constraints and the healthcare system structure can exacerbate these issues. Limited resources may lead to longer preoperative stays due to delays in surgical scheduling or inadequate preoperative optimization, increasing the risk of complications. Moreover, variations in institutional practices, such as the implementation of enhanced recovery protocols, can influence postoperative LOS and outcomes [11]. Few studies address the preoperative LOS and the occurrence of postoperative complications [3, 4, 5, 7, 8, 12, 13]. However, the results are conflicting regarding the increased risks of postoperative complications, although the financial advantage of a shorter hospital stay is obvious. No such study has yet been done in Nepal, which has a different social and demographic stratum. This study aims to evaluate the postoperative complications of neurosurgical patients who underwent elective neurosurgery in a large university hospital in Nepal. We aim to determine the correlation between pre‐operative LOS and postoperative complications.

## 2 Materials and Methods

This retrospective observational study was conducted in the Department of Neurosurgery at Tribhuvan University Teaching Hospital (TUTH), Kathmandu, Nepal. Ethical approval was obtained from the Institutional Review Committee of the Institute of Medicine (approval no: 238(6‐11)E2, 080/081). The requirement for individual informed consent was waived due to the retrospective design, in accordance with institutional and national guidelines.

### 2.1 Study Variables and Definitions

LOS was defined as:

- Preoperative LOS: Days from hospital admission to the day of surgery.
- Total LOS: Days from hospital admission to discharge.

Prespecified analyses included comparisons of complication rates across dichotomized preoperative and total hospital stay [14].

- Preoperative LOS: ≤ 6 days vs. > 6 days.
- Total LOS: ≤ 13 days vs. > 13 days.

### 2.2 Statistical Analysis

Descriptive statistics were used to summarize baseline characteristics. Categorical variables were expressed as counts and percentages, and continuous variables were presented as means with standard deviations (SD) for normally distributed data and medians with interquartile ranges (IQR) for non‐normally distributed data, following the recommendations of the SAMPL (Statistical Analyses and Methods in the Published Literature) guidelines, Altman & Bland, BMJ 1995 [15] and the Guidelines for Reporting of Statistics for Clinical Research in Urology, Assel et al., 2018 [16]. Associations between categorical variables were assessed using the Chi‐square test or Fisher's exact test when appropriate. Binary logistic regression was performed to evaluate the association between preoperative hospital stay and postoperative complications while adjusting for age, sex, and comorbidities. Odds ratios (OR) were reported with 95% confidence intervals (CI). All statistical tests were two‐sided, and a *p*‐value < 0.05 was considered statistically significant. All statistical tests were two‐sided with an *α* of 0.05. Analyses were conducted using SPSS version 26 (IBM Corp., Armonk, NY, USA). This study was conducted in accordance with the STROCSS criteria, ensuring methodological rigor and transparency [17].

Inclusion criteria:

1. All the elective neurosurgical procedures between June 2022 and June 2023.

Exclusion criteria:

1. Emergency surgeries, cases with incomplete medical records, and patients discharged against medical advice.

## 3 Results

One hundred and ten patients who underwent elective neurosurgical procedures during the study period were analyzed. Of the 110 patients, 60 (54.5%) were male and 50 (45.5%) were female. The patients were from a spectrum of age groups (2 months–73 years), with a median of 35 years (interquartile range of 41.5 years). As depicted in Figure 1, patients were from various parts of Nepal, and only one patient was from India.

**FIGURE 1:**
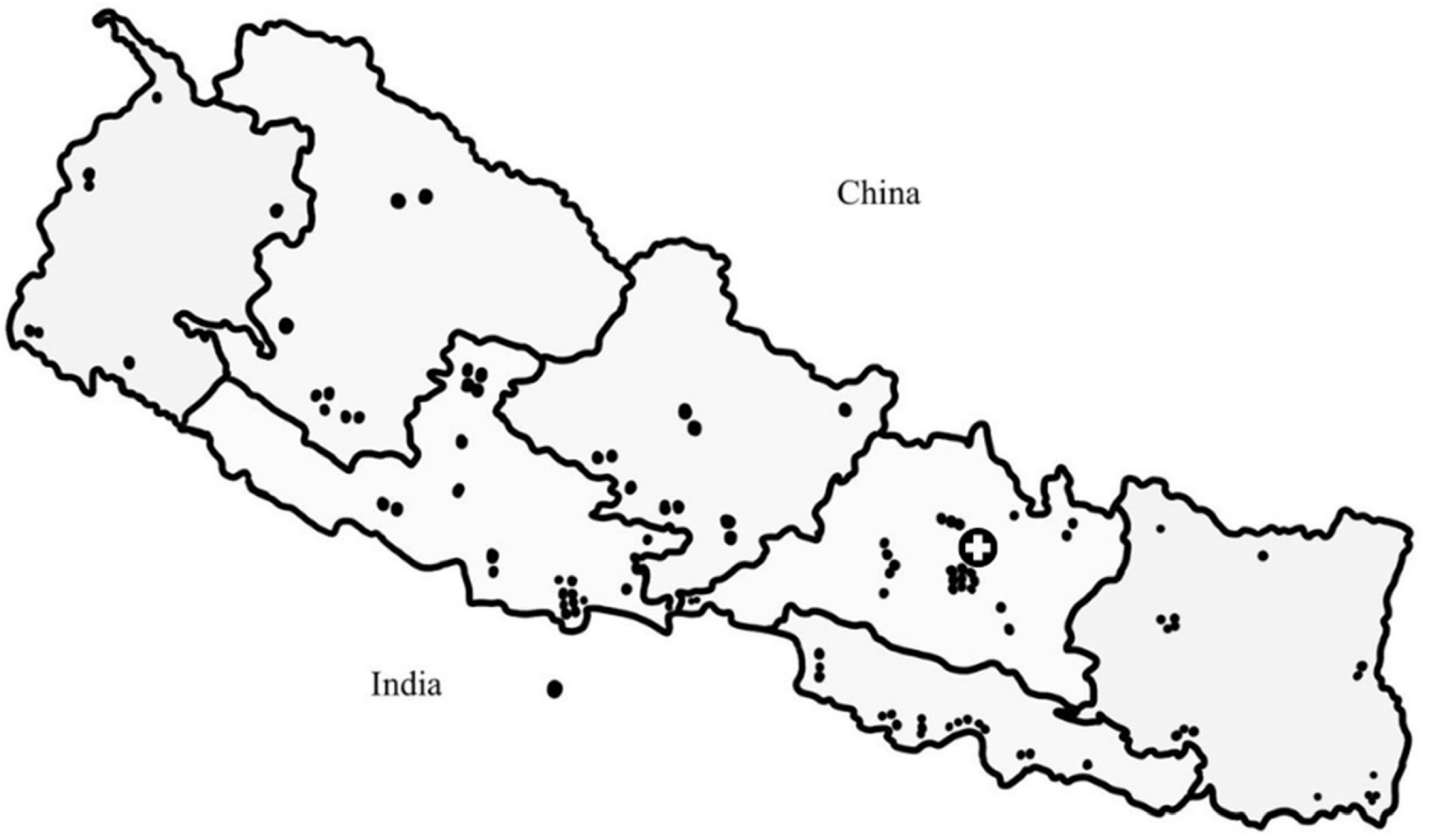
The geographical distribution of patients (black dots) is plotted on the Nepal map. The location of Tribhuvan University Teaching Hospital is denoted as “+.”

The preoperative diagnosis of our patient population is shown in Figure 2. Central nervous system (CNS) tumors comprised 54.5% (60/110) of the elective operative workload, followed by spinal pathologies other than tumors, 15.45% (17/110), such as meningomyelocele (MMC), lipomeningomyelocele, disc diseases, and tethered cord. Vascular diseases 10% (11/110) included arteriovenous malformation (AVM) and aneurysms, whereas infections covered abscesses. There were 7.27% (8/110) cases of congenital hydrocephalus and 1.81% (2/110) cases of neurosurgical infection. Other pathologies 10.9% (12/110) included ventriculoperitoneal shunt malfunction and arachnoid cyst.

**FIGURE 2:**
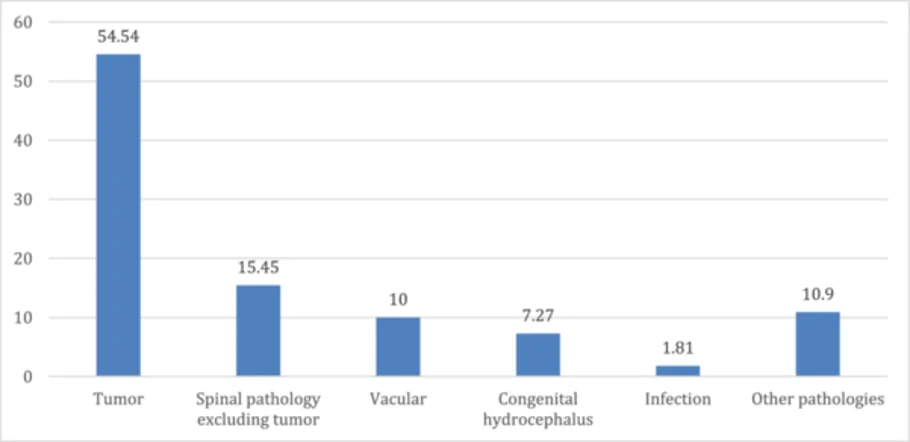
Bar diagram showing percentages of the cases operated.

A total of 78.2% (86/110) of patients had no comorbidities. Among those with comorbidities, 21.8% (24/110), hypertension was by far the most common, 10.9% (12/110), followed by hypothyroidism, 6.4% (7/110), and type 2 diabetes mellitus, 5.5% (6/110). However, no statistically significant relationship was found between the presence of comorbidities and the occurrence of postoperative complications (*p* = 0.133) (Table 1).

**TABLE 1:** Cross‐tabulation and non‐parametric Chi‐square test between the presence of comorbidities and postoperative complications.

Comorbidities	Postoperative complications			
		Yes	No	Total
	Yes	5	19	24
	No	32	54	86
Total		37	73	110
	Value	*df*	Asymptotic significance (two‐sided)	
Pearson chi‐square	2.254	1	0.13	

The median duration of preoperative LOS was 5.5 days with an interquartile range of 7, and the median total LOS was 13 days with an interquartile range of 11 days. Eighty percent (88/110) of our patients had at least 1 day of ICU stay. Neurovascular cases were found to have the most number of pre‐operative and total duration of stays (Table 2).

**TABLE 2:** Number of pre‐op admission days and total duration of stay according to admission diagnosis.

Diagnosis	Median days of admission before the procedure	Median total hospital days
Tumors	6.0	14.5
Congenital hydrocephalus	3.5	8.00
Vascular	9.0	18.0
Spinal pathology, excluding tumor	3.0	9.00
Infection	2.0	16.0
Other pathologies	6.5	11.0
Total	7 (IQR = 7)	13 (IQR = 11)

Abbreviation: IQR, interquartile range.

Out of 110 patients, 34 (30.9%) had postoperative complications, 20% (22/110) had grade 2 of the Clavien–Dindo grading, and 10.9% (12/110) had grade 3 postoperative complications. After surgery, 80.0% (88/110) of patients were transferred to the ICU for close neuromonitoring and elective ventilation. Patients with a preoperative stay of > 6 days had a higher rate of complications (41.3%, 19/46) compared to those with ≤ 6 days (23.4%, 15/64). This association was statistically significant (*p* = 0.045, Chi‐square test). Also, the total days of hospital stay were statistically associated with the presence of postoperative complications (*p* < 0.001) (Table 3).

**TABLE 3:** Frequency table of preoperative and total days with post‐operative complications.

Days of stay	Postoperative complications		Total	*p*‐value
Preoperative days	Yes	No		0.045
< =6	15	49	64	
> 6	19	27	46	
Total days	Postoperative complications		Total	*p*‐value
	Yes	No		< 0.001
< =13	7	49	56	
> 13	27	27	54	

The relationship between the complication grades and the preoperative stay duration was not statistically significant (*p* = 0.063). However, the total hospital stays and grades of the complications showed a statistically significant association (*p* < 0.001) (Table 4).

**TABLE 4:** Frequency table of preoperative and total days with grades of Clavien–Dindo classification.

Days of stay		If yes, under which Clavien–Dindo classification does it fall?			Total	*p*‐value
		Grade I	Grade II	Grade III		
Preoperative days	< =6	49	8	7	64	0.063
	> 6	27	14	5	46	
Total hospital days	< =13	49	4	3	56	< 0.001
	> 13	27	18	9	54	
Total		76	22	12	110	

Binary logistic regression was conducted to assess the relationship between postoperative complications and preoperative hospital stay while adjusting for age, sex, and comorbidities. In logistic regression, preoperative stay showed no statistically significant association with postoperative complications (OR = 1.08, 95% CI 0.854–1.004, *p* = 0.062), indicating that an increase in preoperative hospital stay did not demonstrate a statistically significant impact. Similarly, age (OR = 1.02, 95% CI 0.959–1.001, *p* = 0.068) and sex (OR = 0.55, 95% CI 0.781–4.202, *p* = 0.166) were not significantly associated with postoperative complications. However, the presence of comorbidities showed a marginal association (OR = 0.31, 95% CI 0.956–10.693, *p* = 0.057), suggesting a potential protective effect, though it did not reach statistical significance (Table 5).

**TABLE 5:** Multivariable logistic regression analysis of factors influencing postoperative complications.

Variables	Estimate	Standard error	Wald	Odds Ratio	*p*‐value
Number of days of admission before the procedure/pre‐operative hospital stay	0.07	0.041	3.48	1.08	0.062
Age	0.02	0.011	3.33	1.02	0.068
Sex (Reference = Female)	−0.59	0.429	1.92	0.55	0.166
Comorbidities (Reference = No)	−1.17	0.614	3.62	0.31	0.057

## 4 Discussion

The preoperative LOS is considered from the day of admission to the day before surgery, while the total LOS comprises the number of days before surgery, the day of surgery, and the days after surgery till discharge and is an important factor for resource utilization and cost‐effectiveness of hospital service [4, 18, 19]. In low‐ and middle‐income countries, longer than usual pre‐surgery hospital stays appear to be the most typical neurosurgical scenario due to the uncertainty of operating days or unexpected development of comorbidities, such as the development of hypertension after admission or infection in patients with diabetes [4, 7]. Also, extended hospital stays before surgery could indicate a more severe level of disease and a higher prevalence of comorbid illnesses in surgical patients. The typical length of hospital stay for neurosurgical cases varies significantly depending on the type of surgery and patient‐specific factors. According to the literature, the median length of hospital stay for neurosurgical patients can range from a few days to several weeks. For general neurosurgical cases, a study reported a median LOS of 8 days, with a wide range from 1 to 242 days, indicating substantial variability based on the complexity and complications associated with the surgery [20]. Another study found an average LOS of 6 days across various neurosurgical procedures, with factors such as insurance type and patient comorbidities influencing the duration [21]. A study conducted in Pakistan by Khan et al. shows that the mean hospital stays for elective neurosurgical procedures were 6.5 (±5) days. The mean preoperative stay was 4.5 (±4) days [4]. However, A Canadian study showed a mean duration of LOS before surgery of just 1.2 days [5]. Specific types of neurosurgical procedures have different typical lengths of stay. For instance, patients undergoing craniotomy for tumor resection had a median hospital stay of 4 days, with 27.7% of patients staying at least 8 days [22]. In the context of spine surgery, the median LOS varied by procedure: discectomy had a median stay of 0 days, laminectomy 1 day, and posterior instrumented fusion 4 days [23]. For elective meningioma resection, the median LOS was reported to be 6 days, with variations depending on the day of surgery [24]. In cases involving complications such as intraoperative cerebrospinal fluid (CSF) leaks during endoscopic skull base surgery, the median LOS was 4 days, with factors like the extent of surgery and postoperative complications significantly prolonging the stay [25]. Ventriculostomy procedures, often associated with severe conditions like subarachnoid hemorrhage, had a mean LOS of 20.8 days [26]. The median preoperative stay among patients in our center was 5.5 days, and the total duration of stay was 13 days. Most elective surgeries performed in our center were craniotomies for CNS tumors, followed by spinal and shunt‐related procedures. This trend was similar to the series published in Nepal and India [27, 28]. The reason for our longer LOS could be due to a higher proportion of cranial surgeries in our series.

The present series showed a significant male predominance similar to one publication from our hospital and another from Pakistan [4, 29]. On the contrary, a study in the US revealed a lower percentage of males [30]. In our series, the most common comorbidity associated with the patients undergoing neurosurgery was hypertension, similar to the study conducted in Romania [31].

Neurosurgical patients are at high risk of neurological and systemic complications, even in elective procedures. Hemiparesis, cerebral vasospasms, seizures, diminished consciousness, intraparenchymal hematomas, and surgical site infections are the most often reported neurological side effects, whereas nausea and vomiting, hypotension, respiratory distress, and nosocomial infections are examples of systemic consequences [27]. Our study did not demonstrate a correlation between the presence of co‐morbidity and the incidence of postoperative complications. However, preoperative comorbidities (ASA Score > 3–4) were significantly associated with the occurrence of complications in the study reported by Boissonneau et al. [32]. When comparing the global trends with our study, it is reasonable to assume that other factors were stronger driving forces for developing complications compared to co‐morbidities.

The incidence of postoperative complications in our series was 30.9%, which is similar (35.15%) to the study by Sharma et al. performed in another tertiary center in Kathmandu in 2021 [27]. In contrast, the results reported by Boissonneau et al. in the French population showed a lower incidence of complications (21.6%) [32]. This might be due to a different patient population, their early access to the facilities, or less hospital‐to‐intervention time. A cohort study by Cediel et al. also showed an increased risk of SSI in patients with more extended pre‐operative hospital stays [30]. A significant statistical association between the total duration of hospital stay and complications was expected, as complications lengthened the duration of stay. Our study demonstrated that a preoperative stay longer than 6 days was associated with a higher complication rate (41.3% vs. 23.4%, *p* = 0.045), and a total hospital stay longer than 13 days was strongly associated with postoperative complications (50.0% vs. 12.5%, *p* < 0.001). While these findings were statistically significant, the effect sizes suggest clinical importance as well.

The overall mortality rates following elective and emergency neurosurgery differ significantly. Elective neurosurgical procedures generally have lower mortality rates compared to emergency procedures. For instance, in England, the in‐hospital mortality rates following elective cranial and spinal surgeries are reported to be 0.5% and 0.1%, respectively, whereas non‐elective cranial and spinal surgeries have mortality rates of 7.4% and 1.3% [33]. Similarly, a study on elderly patients undergoing elective craniotomies found a 30‐day mortality rate of 0.55%, while another study reported a 30‐day mortality rate of 0.8% for elective procedures and 10.1% for emergency procedures in patients aged ≥ 70 years [12, 34]. The overall mortality rate following elective neurosurgery is under 1%, while the rate following emergency neurosurgery is 29% [33]. Both groups' risk of death is elevated by postoperative complications [31]. In our case, there was no mortality; most fell under CD grades 2 and 3.

The determinants affecting preoperative and total LOS for neurosurgical cases in Nepal and other low socio‐economic countries are multifaceted and influenced by several factors, as highlighted in the medical literature. In low‐income countries, financial limitations significantly impact the quality and timeliness of neurosurgical care. For instance, compromises such as the use of low‐resolution imaging and limited intraoperative monitoring are common to reduce costs, which can affect preoperative planning and postoperative recovery, potentially prolonging LOS [35]. The type of healthcare system, whether single‐payer or mixed‐payer, influences LOS. Studies indicate that countries with single‐payer systems tend to have longer LOS compared to those with mixed‐payer systems, which may be due to differences in resource allocation and efficiency [13]. Delays in surgical intervention are more pronounced in low‐ and middle‐income countries (LMICs) compared to high‐income countries (HICs). Longer wait times for surgery can lead to worsened preoperative conditions, thereby extending the total LOS [13, 36]. Factors such as the patient's functional status, the need for occupational therapy, and the incidence of hospital‐acquired infections are associated with prolonged LOS. These factors highlight the importance of preoperative preparation and postoperative care in managing LOS [9]. The availability of specialized care, such as dedicated neurocritical ICUs, is often limited in low‐income settings, which can affect both preoperative and postoperative care, thereby influencing LOS [35]. Prior studies have associated longer stays with higher healthcare costs and lower patient satisfaction [37, 38].

## 5 Limitations

This study has certain limitations. First, the sample size of 110 patients from a single university hospital may not be fully representative of the broader population, which limits the generalizability of our findings. A larger multicenter study would be beneficial to enhance the external validity of our results. Additionally, our study is subject to selection bias and potential residual confounding factors. We acknowledge that variables such as comorbidities (e.g., hypertension, diabetes, etc.) and unforeseen surgical delays may have influenced the duration of preoperative hospital stay and, subsequently, postoperative outcomes. However, due to limitations in data availability, these factors were not fully accounted for in our analysis. We recommend future studies to include larger, multi‐center datasets and incorporate a more comprehensive set of confounding factors to validate and generalize our findings.

## 6 Conclusion

Our study identified a significant association between preoperative hospital stay and the occurrence of postoperative complications in elective neurosurgical procedures. While the direct relationship between preoperative stay duration and complication severity showed a trend toward significance, that is, was statistically insignificant, the total length of hospital stay was strongly correlated with the severity of postoperative complications, as classified by the Clavien–Dindo grading system. These findings underscore the potential impact of preoperative hospitalization on surgical outcomes, suggesting that optimizing preoperative management strategies may help reduce postoperative morbidity. Given Nepal's developing economy and challenging geographic terrain, which can contribute to increased lengths of stay and associated costs, further research is warranted to explore the underlying factors influencing these associations and to refine perioperative care protocols in neurosurgical patients. A larger prospective study is recommended to analyze the relationship between preoperative LOS and postoperative complications more comprehensively.

## Author Contributions

**Mohan Raj Sharma:** conceptualization, methodology, software, data curation, investigation, validation, formal analysis, supervision, visualization, project administration, writing – review and editing, writing – original draft, resources. **Ishan Sharma:** conceptualization, methodology, software, data curation, investigation, validation, formal analysis, supervision, visualization, project administration, resources, writing – review and editing, writing – original draft. **Susmin Karki:** conceptualization, methodology, writing – review and editing, writing – original draft, resources, supervision, project administration, visualization, formal analysis, validation, software, data curation, investigation. **Nirav Ojha:** conceptualization, methodology, software, data curation, investigation, validation, formal analysis, supervision, visualization, project administration, resources, writing – review and editing, writing – original draft. **Khusbu Kumari:** conceptualization, methodology, software, data curation, investigation, validation, formal analysis, supervision, visualization, project administration, resources, writing – original draft, writing – review and editing. **Prakash Regmi:** visualization, resources, writing – review and editing, writing – original draft. **Deepak Raj Joshi:** software, formal analysis, supervision, writing – original draft, writing – review and editing. **Gopal Sedain:** conceptualization, writing – review and editing, writing – original draft, supervision. **Sushil Krishna Shilpakar:** conceptualization, supervision, writing – original draft, writing – review and editing.

## Funding

The authors have nothing to report.

## Consent

The ethics committee approved the conduct of the research without explicit consent from the participants (IRB: 238 (6‐11) E2.

## Conflicts of Interest

The authors declare no conflicts of interest.

## Transparency Statement

Dr. Susmin affirms that this manuscript is an honest, accurate, and transparent account of the study being reported; that no important aspects of the study have been omitted; and that any discrepancies from the study as planned (and, if relevant, registered) have been explained.

## Data Availability

The data that support the findings of this study are available from the corresponding author upon reasonable request.
